# Improved Growth and Reproductive Performance and Reshaped Gut Microbiota in Jining Grey Goats Through Nubian Crossbreeding

**DOI:** 10.3390/ani16060863

**Published:** 2026-03-10

**Authors:** Jingchao Cao, Huanxiang Li, Yifan Lu, Aowu Wu, Luyu Wang, Tianxu Liu, Zhengxing Lian

**Affiliations:** Beijing Key Laboratory for Animal Genetic Improvement, National Engineering Laboratory for Animal Breeding, Key Laboratory of Animal Genetics and Breeding of the Ministry of Agriculture, College of Animal Science and Technology, China Agricultural University, Beijing 100193, China

**Keywords:** Jining Grey goat, Nubian, crossbreeding, growth performance, gut microbiota

## Abstract

Crossbreeding indigenous goats with exotic meat breeds is a common strategy to improve production performance, but its combined effects on growth and reproductive traits and the potential contribution of the gut microbiota have not been systematically evaluated in Chinese goat populations. This study used high-prolific Jining Grey (JJ) does mated with Nubian (N) bucks to establish three genetic groups: purebred Jining Grey (JJ), F1 Nubian × Jining Grey (NJ), and backcross offspring (Nubian × (Nubian × Jining Grey); NJJ). Body weight from birth to 12 months and average daily gain were recorded, reproductive traits (lambing rate, litter size, and kid survival) of primiparous and multiparous does were analyzed, and litter total birth weight and weaning weight per lambing were calculated. Fecal samples from JJ and NJJ goats were subjected to 16S rRNA gene sequencing to characterize gut bacterial communities. Body weight differed significantly among the three genetic groups at all ages (*p* < 0.01). Crossbred kids (NJ and NJJ) were about 30% heavier at birth and showed higher body weight and average daily gain during pre- and post-weaning periods than JJ kids (*p* < 0.05), representing a consistent growth advantage. In contrast, lambing rate, litter size and kid survival did not differ among genotypes (*p* > 0.05), whereas litter total birth weight and weaning weight were higher in crossbred than in purebred does (*p* < 0.05). Alpha-diversity indices were similar between JJ and NJJ goats (*p* > 0.05), but community structure was significantly distinct (PERMANOVA, *p* = 0.001), characterized by an increased Firmicutes-to-Bacteroidota ratio and shifts in several energy metabolism-related genera (*p* < 0.05). In conclusion, introgression of Nubian genetics into Jining Grey goats improves growth performance and litter weight while maintaining high prolificacy; these improvements are accompanied by a restructured gut microbiota that is associated with these growth patterns.

## Simple Summary

Jining Grey is a Chinese indigenous goat breed known for its high prolificacy but relatively low growth rate. To improve growth performance while maintaining prolificacy, we crossed Jining Grey does with Nubian bucks and compared purebred and crossbred goats. Crossbred kids were heavier at birth and grew faster from birth to one year of age, while lambing rate, litter size and kid survival remained unchanged. However, total litter birth weight and weaning weight were higher in crossbred does. These findings indicate that shifts in the gut microbiota are associated with variations in growth and reproductive output in crossbred goats, offering a potential microbial link to the heterosis effects.

## 1. Introduction

Growth and reproductive performance in goats are directly related to farm profitability and to meat and milk yield, so they are key traits in animal breeding and production. Crossbreeding is an important strategy to improve livestock performance and has been widely used in goats and other ruminants [1,2]. By crossing different breeds, producers can exploit heterosis so that the offspring exceed the mid-parent level in growth rate, body size, and reproductive capacity [3,4]. Previous studies demonstrate that crossbreeding local goats with high-producing exotic breeds often increases growth rate and body weight gain in the offspring, and may also improve reproductive performance [5]. For example, in a cross between Bangladeshi Black goats and Jamunapari goats, F1 crossbred kids had higher birth weight, fattening gain and litter size than the local dams, with more than 15% heterosis [5,6]. These results indicate that appropriate crossbreeding schemes can improve growth and reproduction in goats at the same time.

Jining Grey goats are a Chinese local breed that is well known for their very high prolificacy. They exhibit early sexual maturity, year-round estrus and an extremely high kidding rate [7]. The average litter size can reach about 2.94 kids per kidding, which is much higher than in many other goat breeds. However, Jining Grey goats have a relatively small body size, with a lower growth rate and meat production. In contrast, Nubian (Anglo-Nubian) goats are a large dual-purpose meat and dairy breed that originated in the United Kingdom. They are highly adaptable, and adult bucks can weigh more than 100 kg, showing strong potential for both meat and milk production [8]. Crossbreeding Jining Grey goats with Nubian goats is therefore expected to combine the high prolificacy of the former with the rapid growth and high meat yield of the latter, so that the offspring may be both highly prolific and good meat producers. The mechanisms underlying these crossbreeding effects on growth and reproduction remain a research focus [9]. While genetic complementation and heterosis typically drive improved growth performance in crossbred offspring —often through enhanced metabolic status and feed conversion efficiency —crossbreeding effects on reproductive traits are more complex. For instance, in West African Sahelian × Nubian crosses, F1 animals showed 40% higher body weight at 24 months, whereas the transmission of prolificacy from local breeds to F1 generations varies significantly. The high prolificacy of local breeds is often partially transmitted to F1 offspring; some studies report increased litter sizes or breeding frequencies compared to parents with lower prolificacy [5,9]. Crossbred offspring may inherit high-prolificacy maternal genes and large-body-size paternal traits, potentially improving kid survival and vigour [10]. However, because heterosis for reproductive traits varies by cross combination, these effects require empirical evaluation.

In recent years, the gut microbiota has been increasingly regarded as an important factor influencing livestock performance and is associated with phenotypic differences [11,12,13]. While rumen microbes are central to fermentation, the hindgut microbiota—analyzed here via fecal profiling—critically contributes to nutrient absorption and host health [14,15,16]. Because of differences in genetic background, goat breeds show marked variation in the structure and composition of their intestinal microbiota, which in turn is linked to the efficiency of nutrient digestion and utilization [17,18]. Analyzing fecal microbial shifts can thus provide insights into the mechanisms underlying growth and reproductive variation. For instance, more feed-efficient goat breeds often exhibit distinct microbial profiles enriched in fibre-degrading taxa. Thus, comparisons of rumen microbiota between Egyptian Zaraibi and Shami goats showed that the more feed-efficient breed had higher abundance of fibre-degrading bacteria and better growth performance. In addition, the gut microbiota may influence reproductive function through effects on metabolic hormones and immune status [19]. In sows, increasing dietary fibre levels altered the gut microbiota during gestation and was associated with marked changes in reproductive hormones such as estrogens [19], suggesting a link between microbes and reproductive physiology. Consequently, characterizing the offspring hindgut microbiota may clarify the biological pathways through which crossbreeding influences production traits.

Taken together, crossbreeding Jining Grey goats with Nubian goats has clear application potential, but the effects on offspring phenotypes—especially growth, reproduction and the role of the gut microbiota—are still not fully understood. The present study aimed to evaluate growth performance and reproductive traits among JJ, NJ, and NJJ goats, while further characterizing the fecal microbiota of JJ and NJJ cohorts to explore microbial associations with heterosis. By integrating phenotypic data with microbial diversity and composition, we sought to evaluate the effectiveness of this crossbreeding scheme and to explore potential mechanisms by which crossbreeding improves production performance in goats, providing a scientific basis for the use and optimization of Jining Grey × Nubian crosses.

## 2. Materials and Methods

### 2.1. Animals, Ethics, and Experimental Design

All animal procedures were approved by the Animal Care and Use Committee of China Agricultural University (No. AW01104202-1-1). This study was carried out at the Jining Grey (Jining Grey) conservation and breeding base (Shan County, Heze, Shandong, China), where the experimental goats were purchased, under uniform housing, feeding, and health management. Three cohorts were enrolled: purebred Jining Grey (JJ), F1 crossbreds (Nubian ♂ × Jining Grey ♀; NJ), and backcross offspring (Nubian × (Nubian × Jining Grey); NJJ). All animals were housed in semi-open pens, fed a standard total mixed ration (TMR) formulated to meet NRC requirements for goats, and had ad libitum access to water. Body weight (BW) was recorded at birth, weaning (105 d), and 6, 8, and 12 months; linear body measurements included withers height (WH), body length (BL), chest girth (CG), and cannon bone circumference (CC). Reproductive records comprised kidding rate, total litter size (TLS), liveborn litter size (LLS), weaned litter size (WLS), litter birth weight, and litter weaning weight; parity was classified as primiparous or multiparous.

A total of 200 goats were enrolled for growth performance monitoring. The sample sizes (*n*) for each group and sex at specific stages were as follows: at birth, JJ (male *n* = 30, female *n* = 30), NJ (male *n* = 27, female *n* = 32), and NJJ (male *n* = 42, female *n* = 41); at weaning, JJ (male *n* = 36, female *n* = 34), NJ (male *n* = 17, female *n* = 21), and NJJ (male *n* = 39, female *n* = 22). For long-term growth monitoring, subset populations were maintained at 8 and 12 months: JJ (male *n* = 12 and 3, female *n* = 20 and 29), NJ (male *n* = 20 and 6, female *n* = 31 and 23), and NJJ (male *n* = 11 and 6, female *n* = 32 and 23).

### 2.2. Phenotyping of Growth and Reproduction

BW and linear measurements (WH, BL, CG, and CC) were recorded by the same trained operator between 09:00 and 11:00 using a calibrated platform scale and anthropometric tape to reduce diurnal and operator variability. Reproductive performance indicators (kidding rate, TLS, LLS, and WLS) were extracted from farm digital records and cross-checked against kidding-season logs; incomplete parities and ambiguous dam–kid links were excluded a priori. For microbiome analysis, fecal samples were collected from 20 non-pregnant, non-lactating adult does (approximately 2 years of age; *n* = 10 per group for JJ and NJJ) that were frequency-matched for age and management to minimize physiological interference. Fecal sampling was selected as a non-invasive proxy to represent the hindgut microbiota, which significantly contributes to nutrient absorption in goats.

### 2.3. Fecal DNA Extraction, 16S Library Preparation, and Sequencing

Genomic DNA was extracted using the QIAamp DNA Stool Mini Kit (QIAGEN, Hilden, Germany) following the manufacturer’s instructions. DNA quality was assessed by agarose gel electrophoresis and Qubit 2.0 fluorometry according to the service provider’s standard procedures. The bacterial 16S rRNA gene V3–V4 region was amplified with primers 341F (CCTACGGGNGGCWGCAG) and 806R (GACTACHVGGGTATCTAATCC) with inline barcodes; no-template controls were included per batch. Amplicons were purified, pooled equimolarly, and submitted to Novogene Co., Ltd. (Tianjin, China) for library preparation and sequencing on an Illumina sequencing platform (Illumina, San Diego, CA, USA) under standard operating procedures. Demultiplexed FASTQ files were processed in QIIME 2, denoised to amplicon sequence variants (ASVs) with DADA2, and taxonomically assigned against SILVA r138; feature tables and taxonomy were exported for downstream statistics.cs.

### 2.4. Bioinformatics and Statistical Analysis

Alpha diversity (Chao1, Shannon, and Simpson) and beta diversity (Jaccard, Bray–Curtis, and UniFrac) were computed in QIIME 2 (version 2023.2); community separation was visualized by principal coordinates analysis (PCoA) and non-metric multidimensional scaling (NMDS) and tested by permutational multivariate analysis of variance (PERMANOVA; 9999 permutations) using the vegan package (version 2.6-4) in R (version 4.2.2). Taxonomic differences were identified by LEfSe (version 1.1.2; LDA > 3.0, *p* < 0.05) and validated using ANCOM-BC (version 2.2.0). The betadisper test in vegan (version 2.6-4) was used to confirm homogeneity of multivariate dispersion. Growth traits (body weight and average daily gain) were analyzed using a Linear Mixed Model (LMM) for repeated measures in IBM SPSS Statistics for Windows (version 26.0; IBM Corp., Armonk, NY, USA) to account for the temporal correlation of data within the same individual. The model included genotype, sex, time (age or period), and their interactions as fixed effects, with individual animal as a random subject effect. Data are presented as least squares means (LSM) ± standard error (SE). Differences between genotypes at each time point were assessed using pairwise comparisons with Bonferroni adjustment. Reproductive traits and body measurements were compared using one-way ANOVA with Tukey’s post hoc test or the Kruskal–Wallis test with Dunn’s adjustment in IBM SPSS Statistics for Windows (version 26.0; IBM Corp., Armonk, NY, USA). All *p*-values were FDR-corrected, and significance was set at α = 0.05. A retrospective power analysis for reproductive traits was conducted in IBM SPSS Statistics for Windows (version 26.0; IBM Corp., Armonk, NY, USA).

## 3. Results

### 3.1. Growth Performance of Jining Grey Goats and Crossbred Offspring

At all observation ages between birth and 12 months, body weight differed significantly among the three genetic groups (*p* < 0.01; Table 1). Crossbred kids containing Nubian ancestry (NJ and NJJ) were consistently heavier than purebred JJ in both sexes; for example, newborn weights (NW) of JJ, NJ and NJJ male kids were 2.03, 2.67 and 2.65 kg, respectively, and at 12 months they reached 23.43, 28.33 and 27.07 kg, with a similar pattern in females. Average daily gain (ADG) also reflected this growth advantage (Table 2). All genotypes showed a typical growth curve with rapid pre-weaning gain followed by slower post-weaning growth. Notably, NJ and NJJ generally had higher ADG than JJ in key growth phases, particularly from 0 to 105 d and during 240–360 d, although the superiority of NJJ was less pronounced in certain phases compared to NJ. Overall, these results indicate that mating Jining Grey does with Nubian bucks substantially increases ADG and final body weight while maintaining the general growth pattern of Jining Grey goats.

### 3.2. Reproductive Performance of Primiparous and Multiparous Ewes

In primiparous ewes, reproductive performance was high and did not differ significantly among the three genetic groups (Table 3). The mean ALB of JJ, NJ and NJJ ewes was 1.81, 1.82 and 1.87, respectively, and ALBL ranged from 1.65 to 1.74 lambs per ewe (*p* > 0.05). Stillbirth rates (SR) remained low across groups (6.98–10.34%), with no significant differences detected (*p* = 0.27). The proportions of single, twin and triplet-or-more litters were also comparable among genotypes. Maternal rearing ability did not differ significantly either: AWL ranged from 1.47 to 1.61 lambs per ewe, PWM of live-born lambs was below 11% in all groups, and OMR ranged from 13.95% to 19.35%, indicating that under the current experimental conditions, primiparous ewes of all three genetic groups maintained comparable reproductive and survival performance, with no statistically significant differences observed.

In multiparous ewes, reproductive capacity further increased, with ALB exceeding 2.4 in all genetic groups (Table 4). ALB values for JJ, NJ and NJJ ewes were 2.54, 2.49 and 2.47, respectively, and ALBL ranged from 2.39 to 2.41, with no significant differences among groups (*p* > 0.05). SR in multiparous ewes was consistently low (4.50–5.63%), suggesting comparable and satisfactory neonatal viability across genotypes. AWL was approximately 2.25–2.27 lambs per ewe, PWM ranged from 4.03% to 5.97%, and OMR from 8.92% to 11.27%, again with no significant genotype effects. Overall, these results suggest that the introgression of Nubian genetics (in F1 and backcross generations) does not significantly impair the high prolificacy or maternal ability of Jining Grey goats within the scope of this study.

### 3.3. Litter Weight of Jining Grey Goat and Its Hybrid Offspring at Birth and Weaning

Although ALB and lamb survival rates were similar among genetic groups, crossbred ewes showed a clear advantage in offspring growth (Table 5). The average litter birth weight (ALBW) of JJ ewes (4.63 kg) was significantly lower than that of NJ (6.09 kg) and NJJ (6.25 kg) ewes (*p* < 0.01). When stratified by litter size, litter birth weights for single (SLBW), twin (TLBW), triplet (TrLBW) and quadruplet litters (QLBW) were consistently higher in the two crossbred groups than in JJ, with NJ progeny usually exhibiting the greatest litter birth weights. A similar pattern was observed at weaning: the mean ALWW of JJ ewes was 23.91 kg, compared with 27.79 kg and 28.38 kg for NJ and NJJ ewes, respectively (*p* < 0.01). Within each litter size category (SLWW, TLWW, TrLWW and QLWW), crossbred ewes produced higher litter weaning weights than purebred JJ ewes. Taken together, these findings demonstrate that N × J crossbreeding, while maintaining comparable prolificacy and lamb survival, markedly increased litter birth and weaning weights, thereby enhancing the meat output per lambing ewe.

### 3.4. Fecal Microbiota Diversity and Composition

**Figure 1:**
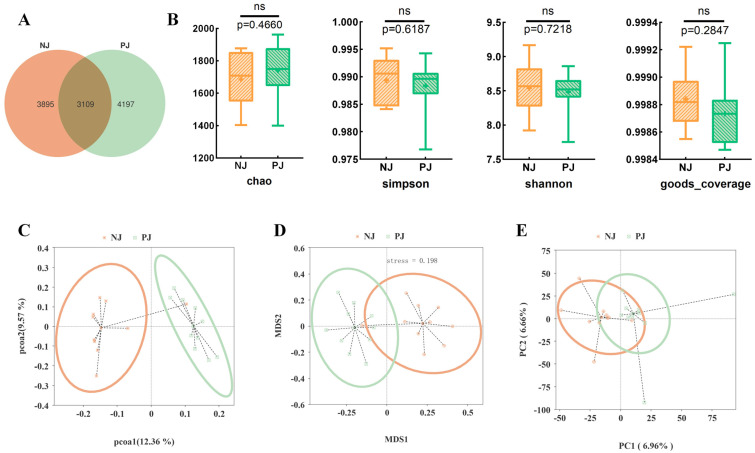
Overall diversity and community structure of fecal microbiota in JJ and NJJ goats. (**A**) Venn diagram showing the numbers of shared and unique ASVs between JJ and NJJ groups (*n* = 10 per group); (**B**) Boxplots of α-diversity indices (Chao1, Simpson, Shannon and Good’s coverage) in the two groups; “ns” indicates no significant difference; (**C**–**E**) Ordination plots (PCoA, NMDS, and PCA). Statistical analysis via PERMANOVA confirmed a significant separation between JJ and NJJ groups (R^2^ = 0.16, *p* = 0.001). Multivariate dispersion was tested using the betadisper function and found to be homogeneous (*p* > 0.05).

**Figure 2:**
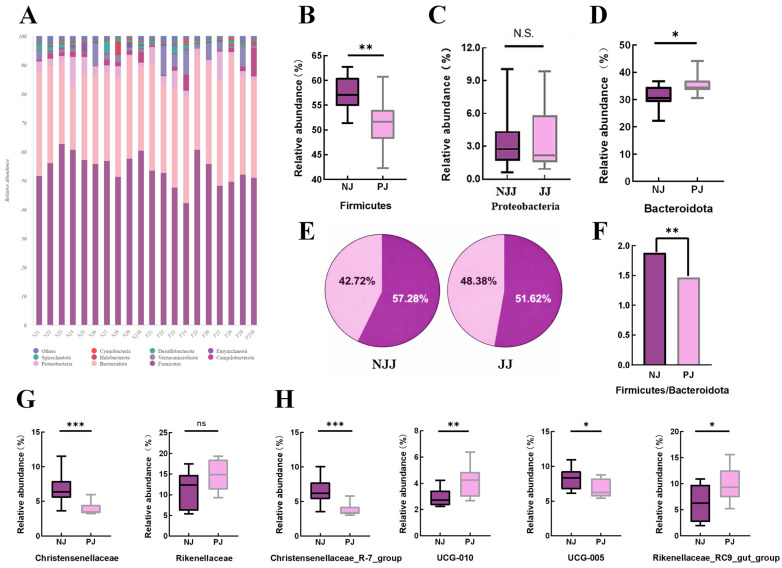
Differences in fecal microbial composition between NJJ and JJ goats. (**A**) Stacked bar plots showing the relative abundance of major bacterial phyla in the feces of NJJ and JJ goats; (**B**–**D**) Boxplots of the relative abundance of Firmicutes, Proteobacteria and the Firmicutes/Bacteroidota ratio, respectively; (**E**) Proportions of Gram-positive (G^+^) and Gram-negative (G^−^) bacteria in NJJ and JJ groups; (**F**) Ratio of G^+^ to G^−^ bacteria; (**G**) Relative abundance of key bacterial families (e.g., Christensenellaceae, Rikenellaceae); (**H**) Relative abundance of representative genera or genus-level groups (Christensenellaceae_R7_group, UCG-010, Rikenellaceae_RC9_gut_group, UCG-005). Data are shown as boxplots (median, interquartile range, and range). *, ** and *** indicate *p* < 0.05, *p* < 0.01 and *p* < 0.001, respectively; ns indicates no significant difference.

**Figure 3:**
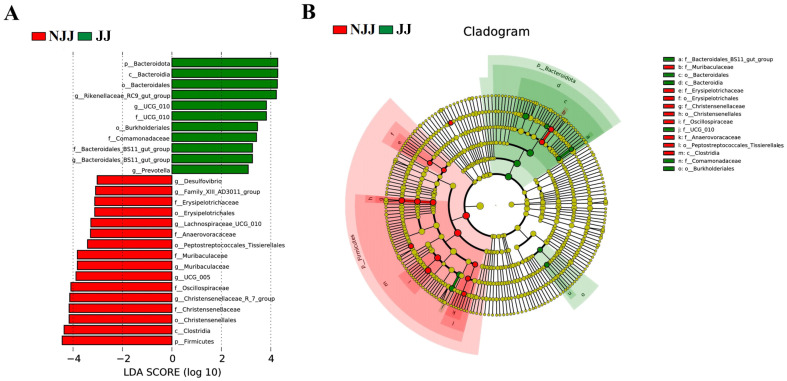
Key bacterial biomarkers differentiating JJ and NJJ genotypes identified by LEfSe analysis. (**A**) Linear discriminant analysis (LDA) scores (log_10_) of bacterial taxa that differed significantly between NJJ (red bars) and JJ (green bars) groups, as identified by LEfSe. (**B**) Cladogram showing the phylogenetic distribution of taxa enriched in each group; coloured circles indicate taxa significantly associated with NJJ (red) or JJ (green) goats, from phylum (outer rings) to genus (inner rings). Only taxa meeting the LEfSe threshold (α = 0.05, LDA score above the preset cutoff) are shown.

High-throughput 16S rRNA sequencing of fecal samples revealed complex and diverse bacterial communities in both JJ and NJJ goats (Figure 1). Good’s coverage exceeded 0.99 across groups, indicating sufficient sequencing depth (Figure 1B). Alpha-diversity (Chao1, Shannon, and Simpson indices) revealed high bacterial richness and diversity, with no significant differences observed between genotypes (*p* > 0.05; Figure 1B). The Venn diagram demonstrated a large core set of shared ASVs together with group-specific ASVs unique to each genotype (Figure 1A). At the taxonomic level, the fecal microbiota of both groups was dominated by Firmicutes, Bacteroidota and Proteobacteria (Figure 2A). While a shift in the Firmicutes-to-Bacteroidota ratio was observed (Figure 2D), we interpret this as a descriptive change in community structure associated with the crossbred genotype rather than a direct functional driver of metabolic efficiency, pending further metagenomic validation. Analysis of community structure (PCoA, NMDS and PCA) based on Jaccard and Bray–Curtis distances revealed a clear separation between the JJ and NJJ samples (Figure 1C–E). This separation was statistically significant (PERMANOVA: R^2^ = 0.16, *p* = 0.001; Figure 1C–E). Crucially, the betadisper test confirmed homogeneous multivariate dispersion (*p* > 0.05), ensuring that group clustering was driven by community shifts rather than within-group heterogeneity. These results confirm that the observed microbial clustering is primarily driven by genotype-associated community shifts rather than within-group variance. At the family and genus levels, several taxa showed clear shifts in relative abundance, including Christensenellaceae and Rikenellaceae, as well as the genera Christensenellaceae_R-7_group, UCG-010, Rikenellaceae_RC9_gut_group and UCG-005 (Figure 2G,H). LEfSe analysis identified these and additional taxa as discriminative biomarkers of the two genotypes (Figure 3). Multiple Christensenellaceae- and Rikenellaceae-related lineages were significantly enriched in one group or the other, and the cladogram highlighted distinct phylogenetic clusters associated with JJ or NJJ goats (Figure 3A,B). Together, these results indicate that crossbreeding is accompanied by marked but targeted shifts in the composition of the fecal microbiota, superimposed on a broadly shared core community.

## 4. Discussion

The substantial growth advantage observed in our crossbred offspring aligns with the well-documented heterosis and breed complementarity resulting from crossing small-framed indigenous breeds with larger exotic meat breeds. Similar improvements in birth weight and pre-weaning growth have been reported in various terminal cross systems, notably the Boer × Murciano-Granadina cross and other indigenous–exotic combinations. To our knowledge, this study provides the first systematic quantification of these growth benefits in the Jining Grey × Nubian cross within a Chinese production context. These findings extend international evidence to a new genetic background, demonstrating that the introgression of Nubian genetics effectively shifts the offspring growth trajectory upward [1]. This performance advantage likely stems from the integration of additive sire-breed effects with heterosis and breed complementarity [20]. Nubian goats are large-framed and are expected to transmit higher growth potential as additive genetic effects, thereby shifting the offspring growth trajectory upward when used as sires [20]. At the same time, heterosis is frequently expressed in crossbreds, especially for early-growth and fitness-related traits [20]. In line with this, terminal cross kids have been reported to grow faster and even reach slaughter targets earlier than purebreds in controlled systems [1,21]. Evidence from other Boer × indigenous cross systems also supports substantial increases in early weights and gains, reinforcing the generality of “large sire × local dam” cross benefits [22]. It should also be noted that the traits measured in this study mainly included body weight and reproductive performance, and we did not directly evaluate feed efficiency. Because crossbred kids usually grow faster and reach higher body weight, it is reasonable to hypothesize that they may also differ in growth efficiency, but this cannot be tested with the current dataset. Future work should include controlled feeding or metabolism trials to compare feed intake, feed conversion ratio, and energy use efficiency among genotypes. In addition, combining these measurements with rumen microbiota composition and functional profiling would help to clarify the metabolic pathways associated with faster growth in crossbred kids, although direct evidence for improved nutrient and energy utilization remains to be established [23].

Notably, Nubian introgression did not compromise the exceptional prolificacy and maternal ability of Jining Grey goats. Multiparous crossbred ewes maintained litter sizes of 2.4–2.5, demonstrating that the high-fertility trait was preserved under our management conditions. These results align with observations in Sahelian × Anglo-Nubian crosses, where appropriate breeding designs enhanced maternal traits without reducing reproductive output [24]. In contrast, Pérez-Baena et al. [1] found in Murciano-Granadina × Boer terminal crosses that reproductive outcomes were slightly poorer when mating occurred during the non-breeding season. This indicates that under suboptimal mating seasons or greater environmental stress, reproductive performance may be more vulnerable in some crossbreeding schemes [1,25]. Compared with that study, our work was conducted in a favourable breeding season and under good management conditions, and all genotypes showed high conception rates and low kid mortality. We therefore did not detect any adverse effect of crossbreeding on reproduction. Critically, the absence of statistical significance in reproductive metrics must be interpreted with caution. Although our findings suggest that Nubian introgression does not fundamentally impair the prolificacy of Jining Grey goats, the relatively small sample size for primiparous does (*n* = 16–23) may limit the statistical power required to detect subtle phenotypic shifts. From a biological perspective, maintaining a litter size above 2.4 in multiparous crossbred does is highly encouraging for production, yet future large-scale studies are required to confirm these trends across diverse environmental conditions. Taken together, these results support the idea that environment and management strongly modulate the effect of crossbreeding on reproductive performance, because thermal/seasonal stress can disrupt endocrine function and fertility in goats [25,26]. This genotype × environment interaction deserves further investigation [26].

On this basis, our data further show that the main advantage of crossbred kids occurred from birth to weaning. Crossbred kids were heavier at birth and maintained a higher average daily gain during the suckling period, whereas differences in growth between genotypes became smaller after weaning. This pattern suggests that the early-life growth advantage reflects both a higher genetic growth potential and a more favourable uterine and lactational environment. Maternal effects are known to contribute substantially to variation in pre-weaning growth traits in goats [27]. The introduction of Nubian genetics may have improved fetal growth and birth weight, giving crossbred kids a higher starting weight. In addition, crossbred dams or granddams may have benefited from genetic complementarity (including maternal heterosis) that enhances milk yield and mothering ability, thereby supporting faster growth of kids during lactation [28,29]. Although we did not directly measure milk yield or milk composition in this study, crossbreeding programmes in Sahelian goats have reported large increases in doe milk yield together with improved pre-weaning growth of kids [30]. Together with evidence that crossbred does can produce higher milk yield during the pre-weaning period [24], it is plausible that maternal heterosis, including potentially improved milk supply and maternal behaviour, contributes to the early growth advantage observed in Jining Grey × Nubian kids, though this remains a hypothesis to be tested [29]. This maternal contribution likely helps crossbred kids establish a body weight lead early in life and shifts the entire growth curve upward, rather than fundamentally changing its shape.

Finally, crossbreeding Jining Grey goats with Nubian goats did not markedly change fecal microbial α-diversity; overall diversity remained high. However, the community composition was clearly shifted. Compared with purebreds, crossbred kids exhibited a higher relative abundance of Firmicutes and a lower abundance of Bacteroidota, leading to an increased Firmicutes-to-Bacteroidota (F/B) ratio. In this study, we present the elevated F/B ratio as a descriptive structural hallmark of the crossbred microbiota rather than a functional driver of feed efficiency. While the Firmicutes-to-Bacteroidota (F/B) ratio is often associated with production phenotypes in ruminants, its interpretation remains inconsistent across the literature [31]. In this study, we present the elevated F/B ratio as a descriptive structural hallmark of the reshaped microbiota rather than a functional driver of feed efficiency. The observed shifts likely represent a community-level response to the altered physiological demands of faster-growing genotypes rather than a primary driver of growth itself; however, this does not provide direct evidence of superior feed efficiency in the absence of intake data [16,32]. This agrees with the study of Shi et al. in Dongshan × Nubian goats, where crossbreeding improved growth and carcass traits and was accompanied by characteristic shifts in rumen microbiota [33]. At the genus/family level, several bacterial groups were closely associated with growth. Christensenellaceae was significantly less abundant in crossbred kids than in purebred Jining Grey kids, which had slower growth. In young goats, a Ruminococcus-dominated rumen cluster enriched in Christensenellaceae R-7 group showed higher growth rate and higher ruminal propionate than a Prevotella-dominated cluster, highlighting that the growth relevance of this family can depend on niche and community context [34]. It is critical to note that fecal microbiota does not always reliably represent rumen microbial communities. Therefore, our fecal 16S profiles should be interpreted strictly as hindgut signals, which may not mirror rumen-trait associations [35]. In our data, bacterial groups involved in carbohydrate fermentation and energy production tended to increase in crossbred kids. Prevotellaceae and succinate-producing Succinivibrionaceae are common fermentation guilds implicated in host energy harvest and propionate-related pathways, although their associations with feed efficiency can be context dependent [16,31]. By contrast, Rikenellaceae_RC9 was more abundant in purebred Jining Grey kids and has been reported to respond to feeding system and forage/roughage levels in ruminants [36]. These taxonomic shifts are associated with faster growth patterns, though they do not provide direct evidence of superior nutrient utilization in the absence of intake data. Future research utilizing metagenomics and targeted metabolomics (e.g., VFAs) is necessary to validate the functional contributions of these candidate taxa to digestion efficiency.

## 5. Conclusions

In conclusion, crossing high-prolific Jining Grey does with Nubian bucks markedly improves the growth performance of their progeny without compromising reproductive efficiency. Crossbred kids are consistently heavier and show higher average daily gain from birth to 12 months of age, and crossbred does produce litters with greater total birth and weaning weights. At the same time, key reproductive traits such as lambing rate, litter size and kid survival remain comparable to those of purebred Jining Grey. The accompanying remodelling of the gut microbiota may partly underlie the observed heterosis in growth and litter weight.

## Figures and Tables

**Table 1:** Least Squares Means (LSM) ± Standard Error for body weight of Jining Grey (JJ) and crossbred (NJ, NJJ) goats at different growth stages.

Items ^1^	JJ (LSM ± SE)	NJ (LSM ± SE)	NJJ (LSM ± SE)	*p*-Value
Male goat				
NW ^2^, kg	2.03 ± 0.07 ^b^	2.67 ± 0.14 ^a^	2.65 ± 0.09 ^a^	<0.01
WW ^3^, kg	11.33 ± 0.26 ^b^	12.94 ± 0.51 ^a^	12.80 ± 0.35 ^a^	<0.01
BW8 ^4^, kg	17.32 ± 0.47 ^b^	20.44 ± 0.69 ^a^	19.25 ± 0.76 ^ab^	<0.01
BW12, kg	23.43 ± 0.56 ^b^	28.33 ± 0.69 ^a^	27.07 ± 0.55 ^a^	<0.01
Female goat				
NW, kg	1.93 ± 0.07 ^b^	2.63 ± 0.10 ^a^	2.51 ± 0.09 ^a^	<0.01
WW, kg	10.50 ± 0.36 ^b^	12.69 ± 0.45 ^a^	11.24 ± 0.40 ^ab^	<0.01
BW8, kg	15.96 ± 0.50 ^b^	18.80 ± 0.60 ^a^	17.64 ± 0.55 ^ab^	<0.01
BW12, kg	19.20 ± 0.70 ^b^	23.80 ± 0.85 ^a^	23.18 ± 0.80 ^a^	<0.01

Note: ^1^ JJ, purebred Jining Grey; NJ, Nubian × Jining Grey F1 hybrids; NJJ, (Nubian × Jining Grey) × Jining Grey backcross offspring; ^2^ NW: Newborn weight; ^3^ WW: Weaning weight; ^4^ BW, Body weight. Data are presented as Least Squares Means (LSM) ± Standard Error (SE). Sample sizes (*n*) vary by age and sex; detailed *n*-values for each group at birth, weaning, 8 months, and 12 months are provided in Section 2.1. Different superscript letters (a, b) within the same row indicate significant differences among genotypes (*p* < 0.05), whereas values sharing at least one common letter are not significantly different. Therefore, values marked with “ab” do not differ significantly from those marked with either “a” or “b”. Sample sizes (*n*) vary by age and sex; detailed *n* values for each group at birth, weaning, 8 months, and 12 months are provided in Section 2.1.

**Table 2:** Least Squares Means (LSM) ± Standard Error for average daily gain (g/d) of Jining Grey and crossbred goats during different growth periods.

Items ^1^	JJ (LSM ± SE)	NJ (LSM ± SE)	NJJ (LSM ± SE)	*p*-Value
Male goat				
0–105 d	87.68 ± 2.40 ^b^	97.70 ± 2.65 ^a^	96.69 ± 2.10 ^a^	<0.01
105d–180 d	39.73 ± 2.00 ^b^	40.69 ± 2.50 ^b^	49.09 ± 2.80 ^a^	<0.01
180d–240 d	51.70 ± 4.30 ^b^	74.15 ± 5.10 ^a^	46.14 ± 4.50 ^b^	<0.01
240d–360 d	50.94 ± 2.60 ^b^	65.78 ± 3.50 ^a^	65.14 ± 3.20 ^a^	<0.01
Female goat				
0–105 d	82.42 ± 3.00 ^b^	98.24 ± 3.20 ^a^	83.18 ± 2.80 ^b^	<0.01
105d–180 d	49.21 ± 1.80 ^a^	40.38 ± 2.10 ^b^	46.06 ± 1.90 ^ab^	<0.01
180d–240 d	28.04 ± 3.90 ^b^	47.00 ± 4.20 ^a^	49.13 ± 4.00 ^a^	<0.01
240d–360 d	27.05 ± 3.30 ^b^	41.65 ± 3.80 ^a^	46.15 ± 3.50 ^a^	<0.01

Note: ^1^ NJ, Nubian × Jining Grey F_1_ crossbreds (N × J); NJJ, backcross progeny from NJ bucks mated to Jining Grey does (NJ × J). Data are presented as Least Squares Means (LSM) ± Standard Error (SE). Sample sizes (*n*) vary by age and sex; detailed *n*-values for each group at birth, weaning, 8 months, and 12 months are provided in Section 2.1. Different superscript letters (a, b) within the same row indicate significant differences among genotypes (*p* < 0.05), whereas values sharing at least one common letter are not significantly different. Therefore, values marked with “ab” do not differ significantly from those marked with either “a” or “b”. Sample sizes (*n*) vary by age and sex; detailed *n* values for each group at birth, weaning, 8 months, and 12 months are provided in Section 2.1.

**Table 3:** Comparison of lambing and survival performance of primiparous ewes among JJ, NJ and NJJ groups.

Items ^1^	JJ	NJ	NJJ	SEM	*p*-Value
ALB ^2^	1.81	1.82	1.87	0.23	0.87
ALBL ^3^	1.73	1.65	1.74	0.18	0.16
AWL ^4^	1.5	1.47	1.61	0.16	0.23
SR ^5^	10.34%	9.68%	6.98%	**-**	0.27
PWM ^6^	7.69%	10.71%	7.50%	**-**	0.32
OMR ^7^	17.24%	19.35%	13.95%	-	0.22

Note: ^1^ NJ, Nubian × Jining Grey F_1_ crossbreds (N × J); NJJ, backcross progeny from NJ bucks mated to Jining Grey does (NJ × J); ^2^ ALB, average litter size (lambs born per ewe); ^3^ ALBL, average number of live-born lambs per ewe; ^4^ AWL, average number of weaned lambs per ewe; ^5^ SR, stillbirth rate (number of stillborn lambs/total lambs born); ^6^ PWM, pre-weaning mortality of live-born lambs (number of lamb deaths before weaning/total live-born lambs); ^7^ OMR, overall lamb mortality (total dead lambs/total lambs born). Sample sizes (*n*) values represent the number of kidding records. For each group, the sample sizes for primiparous/multiparous does are JJ (16/56), NJ (17/63), and NJJ (23/44).

**Table 4:** Comparison of lambing and survival performance of multiparous ewes among JJ, NJ and NJJ groups.

Items ^1^	JJ	NJ	NJJ	SEM	*p*-Value
ALB ^2^	2.54	2.49	2.47	0.13	0.87
ALBL ^3^	2.39	2.37	2.41	0.14	0.56
AWL ^4^	2.25	2.27	2.27	0.11	0.43
SR ^5^	5.63%	5.10%	4.50%	-	0.33
PWM ^6^	5.97%	4.03%	5.67%	-	0.27
OMR ^7^	11.27%	8.92%	9.91%	-	0.41

Note: ^1^ NJ, Nubian × Jining Grey F_1_ crossbreds (N × J); NJJ, backcross progeny from NJ bucks mated to Jining Grey does (NJ × J); ^2^ ALB, average litter size (lambs born per ewe); ^3^ ALBL, average number of live-born lambs per ewe; ^4^ AWL, average number of weaned lambs per ewe; ^5^ SR, stillbirth rate (number of stillborn lambs/total lambs born); ^6^ PWM, pre-weaning mortality of live-born lambs (number of lamb deaths before weaning/total live-born lambs); ^7^ OMR, overall lamb mortality (total dead lambs/total lambs born). Sample sizes (*n*) values represent the number of kidding records. For each group, the sample sizes for primiparous/multiparous does are JJ (16/56), NJ (17/63), and NJJ (23/44).

**Table 5:** The litter weight of Jining Grey goats and their hybrid offspring.

Items ^1^, (kg)	JJ	NJ	NJJ	SEM	*p*-Value
ALBW ^2^	4.63 ^b^	6. 09 ^a^	6.25 ^a^	0.25	<0.01
SLBW ^3^	2.25 ^b^	2.92 ^a^	2.81 ^ab^	0.11	<0.01
TLBW ^4^	4.06 ^b^	5.31 ^a^	5.37 ^a^	0.14	<0.01
TrLBW ^5^	5.51 ^b^	7.54 ^a^	7.46 ^a^	0.17	<0.01
QLBW ^6^	7.79 ^b^	9.62 ^a^	8.35 ^b^	0.33	<0.01
ALWW ^7^	23.91 ^b^	27.79 ^ab^	28.38 ^a^	1.31	0.02
SLWW ^8^	11.84 ^b^	13.55 ^a^	13.32 ^ab^	0.41	<0.01
TLWW ^9^	21.15 ^b^	24.34 ^a^	24.11 ^a^	0.27	<0.01
TrLWW ^10^	31.09 ^b^	36.10 ^a^	35.66 ^a^	0.48	<0.01
QLWW ^11^	40.83 ^b^	47.03 ^a^	45.50 ^ab^	0.49	<0.01

Note: ^1^ NJ, Nubian × Jining Grey F_1_ crossbreds (N × J); NJJ, backcross progeny from NJ bucks mated to Jining Grey does (NJ × J); ^2^ ALBW, average litter birth weight; ^3^ SLBW, litter birth weight of single-lamb litters; ^4^ TLBW, litter birth weight of twin litters; ^5^ TrLBW, litter birth weight of triplet litters; ^6^ QLBW, litter birth weight of quadruplet (≥4) litters; ^7^ ALWW, average litter weaning weight; ^8^ SLWW, litter weaning weight of single-lamb litters; ^9^ TLWW, litter weaning weight of twin litters; ^10^ TrLWW, litter weaning weight of triplet litters; ^11^ QLWW, litter weaning weight of quadruplet (≥4) litters. Different superscript letters (a, b) within the same row indicate significant differences among genotypes (*p* < 0.05), whereas values sharing at least one common letter are not significantly different. Therefore, values marked with “ab” do not differ significantly from those marked with either “a” or “b”. Sample sizes (*n*) vary by age and sex; detailed *n* values for each group at birth, weaning, 8 months, and 12 months are provided in Section 2.1.

## Data Availability

The data presented in this study are available in the article.

## Abbreviations

The following abbreviations are used in this manuscript:

JJ	Jining Grey Goat
NJ	F_1_ Nubian × Jining Grey Crossbred
NJJ	Backcross Nubian × (Nubian × Jining Grey)
BW	Body Weight
WH	Withers Height
BL	Body Length
CG	Chest Girth
CC	Cannon Circumference
ADG	Average Daily Gain
TLS	Total Litter Size
LLS	Liveborn Litter Size
WLS	Weaned Litter Size
ALB	Average Litter Size
ALBL	Average Number of Live-born Lambs per Ewe
AWL	Average Number of Weaned Lambs per Ewe
SR	Stillbirth Rate
PWM	Pre-Weaning Mortality
OMR	Overall Mortality Rate
ALBW	Average Litter Birth Weight
ALWW	Average Litter Weaning Weight
SLBW	Single Litter Birth Weight
TLBW	Twin Litter Birth Weight
TrLBW	Triplet Litter Birth Weight
QLBW	Quadruplet Litter Birth Weight
SLWW	Single Litter Weaning Weight
TLWW	Twin Litter Weaning Weight
TrLWW	Triplet Litter Weaning Weight
QLWW	Quadruplet Litter Weaning Weight
ASV	Operational Taxonomic Unit
PCoA	Principal Coordinates Analysis
NMDS	Non-Metric Multidimensional Scaling
PCA	Principal Component Analysis
LEfSe	Linear Discriminant Analysis Effect Size
LDA	Linear Discriminant Analysis
QIIME	Quantitative Insights Into Microbial Ecology
ASV	Amplicon Sequence Variant
FDR	False Discovery Rate
SOP	Standard Operating Procedure
DNA	Deoxyribonucleic Acid
rRNA	Ribosomal RNA
VFA	Volatile Fatty Acid
PERMANOVA	Permutational Multivariate Analysis of Variance
